# Quorum quenching effect of cyclodextrins on the pyocyanin and pyoverdine production of *Pseudomonas aeruginosa*

**DOI:** 10.1007/s00253-024-13104-7

**Published:** 2024-03-22

**Authors:** Ildikó Fekete-Kertész, Zsófia Berkl, Kata Buda, Éva Fenyvesi, Lajos Szente, Mónika Molnár

**Affiliations:** 1 Budapest University of Technology and Economics, Faculty of Chemical Technology and Biotechnology, Department of Applied Biotechnology and Food Science, Műegyetem rkp. 3., Budapest, H-1111 Hungary; 2 CycloLab Cyclodextrin R&D Laboratory Ltd., Illatos u. 7., Budapest, H-1097 Hungary

**Keywords:** Cyclodextrins, *Pseudomonas aeruginosa*, Pyocyanin, Pyoverdine, Quorum quenching, Quorum sensing

## Abstract

**Abstract:**

Various virulence determinants in *Pseudomonas aeruginosa* are regulated by the quorum sensing (QS) network producing and releasing signalling molecules. Two of these virulence determinants are the pyocyanin and pyoverdine, which interfere with multiple cellular functions during infection. The application of QS-inhibiting agents, such as cyclodextrins (CDs), appears to be a promising approach. Further to method development, this research tested in large-volume test systems the effect of α- and β-CD (ACD, BCD) at 1, 5, and 10 mM concentrations on the production of pyocyanin in the *P. aeruginosa* model system. The concentration and time-dependent quorum quenching effect of native CDs and their derivatives on pyoverdine production was tested in a small-volume high-throughput system. In the large-volume system, both ACD and BCD significantly inhibited pyocyanin production, but ACD to a greater extent. 10 mM ACD resulted in 58% inhibition, while BCD only ~40%. Similarly, ACD was more effective in the inhibition of pyoverdine production; nevertheless, the results of RMANOVA demonstrated the significant efficiency of both ACD and BCD, as well as their derivatives. Both the contact time and the cyclodextrin treatments significantly influenced pyoverdine production. In this case, the inhibitory effect of ACD after 48 h at 12.5 mM was 57%, while the inhibitory effect of BCD and its derivatives was lower than 40%. The high-level significant inhibition of both pyocyanin and pyoverdine production by ACD was detectable. Consequently, the potential value of CDs as QS inhibitors and the antivirulence strategy should be considered.

**Keypoints:**

• *Applicability of a simplified method for quantification of pyocyanin production was demonstrated.*

• *The cyclodextrins significantly affected the pyocyanin and pyoverdine production.*

• *The native ACD exhibited the highest attenuation in pyoverdine production.*

**Supplementary Information:**

The online version contains supplementary material available at 10.1007/s00253-024-13104-7.

## Introduction

In recent years, the exploration of repurposing existing pharmaceutical compounds for novel clinical indications has emerged as a significant focal point within the field of drug discovery (Baldelli et al. 2020). The increasing rates of bacterial resistance to current antibiotics require innovative methods targeting bacterial virulence with potentially weaker selection for resistance than in the case of conventional antibiotics (Haque et al. 2021; Maisetta et al. 2021). Antibiotic-resistant *Pseudomonas aeruginosa* is an emerging clinical challenge (Conway et al. 2003; Buzid et al. 2016; Azam and Khan 2019) characterized by a broad spectrum of potential hosts and pathophysiological aspects of infection with a large arsenal of virulence factors and exoproducts, including elastase, hemolysin, rhamnolipids, pyocyanin, and pyoverdine (Lyczak et al. 2000; Kang et al. 2017). As there is clear evidence of the importance of pyocyanin in the pathogenesis of *P. aeruginosa* strains (Kipnis et al. 2006) therapeutic strategies for treating infections by this organism have been suggested targeting pyocyanin (Garner et al. 2012).

Various virulence factors and secondary metabolites in *Pseudomonas aeruginosa* are controlled through the operation of a hierarchical quorum sensing (QS) system (Diggle et al. 2006). Quorum sensing is the mechanism by which bacteria communicate through the production, detection, and response to low molecular weight compounds, called signal molecules or autoinducers (AI) (Popat et al. 2015; Singh et al. 2015) initially documented in the early 1970s concerning a Gram-negative marine bioluminescent bacterium, *Aliivibrio fischeri* (Nealson et al. 1970). This mechanism enables bacteria to communicate and collaborate in the regulation of gene expression. The QS pathways are complex, including multiple autoinducer signals and receptors (Perez and Hagen 2010).

As much as 6% of all genes are regulated by quorum sensing involved in virulence, pigment production, and biofilm formation of *P. aeruginosa*, the most in-detail-studied quorum sensing pathogen (Hirakawa and Tomita 2013). In this Gram-negative bacterium, the hierarchical quorum sensing system consists of two chemically distinct classes of signal molecules, N-acylhomoserine lactones (AHLs) and 4-quinolones (AHQs) (Buzid et al. 2016). *P. aeruginosa* produces two acyl-homoserine lactone (AHL) signal molecules: 3-oxododecanoyl-homoserine lactone (3-oxo-C12-HSL) and butanoyl-homoserine lactone (C4-HSL) (Pearson et al. 1994, 1995) both generated by AHL synthases. The LasIR QS system is initiated when the bacterial cell density reaches a QS system-specific threshold, activating the RhlIR system (Hirakawa and Tomita 2013). The las and the systems will activate individually or together the production of virulence factors such as pyocyanin (Smith and Iglewski 2003). *P. aeruginosa* also produces a non-AHL signal molecule, 2-heptyl-3-hydroxy-4-quinolone of the *Pseudomonas* quinolone system (PQS) which is interlinked with the AHL system, suggesting that PQS could be also important for virulence of the organism (Huse and Whiteley 2011). The production of pyoverdine, a yellow-green, water-soluble fluorescent pigment, is also regulated by QS. Pyoverdine is a powerful iron(III) scavenger and an efficient iron(III) transporter and has an important physiological function in satisfying the iron requirement of these facultative anaerobic bacteria (Meyer 2000; Schobert and Jahn 2010). In recent years, the multifunctional regulatory role of the PQS system in biofilm development and pyoverdine production has also been discovered (Diggle et al. 2007; Kang et al. 2017; Díaz-Pérez et al. 2022).

The disruption of the QS system in bacteria opens new potential for efficiently overcoming the problem of antibiotic resistance (Bhardwaj et al. 2013; Nafee et al. 2014). The QS process can be modified through various mechanisms, including diminishing the activity of the AHL cognate receptor protein or AHL synthase, obstructing the synthesis of QS signal molecules, and breaking down autoinducers (Kalia and Purohit 2011), and mimicking the signal molecules through the utilization of synthetic compounds designed as analogs of these signalling molecules (Morkunas et al. 2012; Kalia 2013; Lade et al. 2014). The compounds accountable for hindering autoinducer-triggered quorum sensing systems are referred to as quorum-sensing inhibitors (QSIs). In the past decade, there have been several attempts to discover natural and synthetic compounds (chitosan, flavonoids, furanones, ostarine, parthenolide, 3-phenyllactic acid, sitagliptin, ureidothiophene-2-carboxylic acids, etc.) having the ability to inhibit pyocyanin biosynthesis by selectively disrupting or modulating QS (Hodgkinson et al. 2012, Miller et al. 2015; Kalia 2013; El-Shaer et al. 2016; Kalia et al. 2018; Rubini et al. 2019; Abbas et al. 2020; Dong et al*.* 2001; Maisetta et al. 2021).

In recent years, cyclodextrins (CDs) were demonstrated to efficiently modulate bacterial QS in several Gram-negative and Gram-positive model organisms (Fenyvesi and Sohajda 2022; Nguyen et al. 2022). Cyclodextrins (CDs) are cyclic oligosaccharides composed of 6, 7, or 8 glucopyranose moieties (α-, β-, and γ-CD) produced from starch by enzymatic reactions (Szejtli 1998; Szente et al. 2016).

These molecules are characterized by a hydrophilic surface due to the hydroxyl groups at the rim of their rings surrounding a slightly hydrophobic cavity, thus they can host hydrophobic molecules of proper shape and size to form inclusion complexes (Simoes et al. 2015; Crini 2021). Some studies investigated the QQ possibilities of CDs on AI-mediated QS in different bacterial model systems like *P. aeruginosa*, *Serratia marcescens*, *Chromobacterium violaceum*, or *A. fischeri*. Ikeda et al. (2002) were the first to control autoinducer activities and quorum sensing in *P. aeruginosa* by adding cyclodextrin to the bacterial culture medium assuming that it forms inclusion complexes with autoinducers and keeps their available concentrations at a low level. They found that the addition of 10 mM β-CD decreased the abundance of free AHL signal molecules without inhibiting bacterial growth and meanwhile, it reduced the autoinducing activity in the *P. aeruginosa* culture.

Morohoshi et al. (2013) got promising results applying 10 mg/mL 6-alkylamino-β-CD derivatives in the *S. marcescens* prodigiosin pigment production model system. They also observed that β-CD inhibited the violacein production of *C. violaceum* by about 40%. The native β-CD did not exhibit inhibitory activity on elastase production. In contrast, 6-dodecylamino-β-CD and 6,6′-dioctylamino-β-CD induced approximately 70% and 90% inhibition of elastase activity in *P. aeruginosa* (Morohoshi et al. 2013).

Molnár et al. (2021) demonstrated the QQ effect of various cyclodextrin derivatives in the *A. fischeri* model system. Based on the result of their systematic study focusing on bioluminescence with 12 different cyclodextrins, the autoinducer-dependent quorum sensing mechanism was significantly inhibited by several tested CD compounds. Berkl et al. (2022) were the first to demonstrate in a systematic study including 8 different CD derivatives, that CDs can attenuate biofilm formation, a QS-mediated virulence factor of *P. aeruginosa* PAO1. Their results revealed the ability of the α-CD, randomly methylated α- and β-CDs to significantly inhibit biofilm formation in *P. aeruginosa* suggesting their potential superiority in antibiofilm treatments when compared to conventional approaches like antibiotics.

Several studies focused on the effectiveness of QS inhibitors influencing the QS-mediated virulence factors in *P. aeruginosa* (Sepahi et al. 2015; Chatterjee et al. 2017; Hernando-Amado et al. 2020); nevertheless, there is a paucity of information on the QQ effect of CDs, especially on the virulence-determinant pyocyanin and the similarly QS-regulated pyoverdine pigment production. To the best of our knowledge, comprehensive comparative studies involving a broad spectrum of cyclodextrin derivatives conducted in a time- and concentration-dependent manner are lacking; furthermore, studies focusing specifically on autoinducer-dependent pigment (pyoverdine and pyocyanin) production have not been published; however, some results are available on the QQ effect of native CDs, their derivatives, or immobilized engineered systems in *P. aeruginosa* using different endpoints (Ikeda et al. 2002; Kato et al. 2006, 2007; Morohoshi et al. 2013; Barnaby et al. 2019; Ziegler et al. 2021).

Given that the control of QS by CDs is an innovative approach and there is limited information about their effects on bacterial communication-regulated processes, in this paper, we report a systematic research on the time and concentration-dependent QQ effect of α- and β-CD molecules and their derivatives in the *P. aeruginosa* model system as these CD derivatives may interfere with the control mechanisms of pyoverdine and pyocyanin pigment production without affecting directly bacterial viability. Since previous studies have already demonstrated that CDs influence the biofilm formation of *P. aeruginosa* and that both pyocyanin and pyoverdine indirectly modulate the formation of biofilms, we hypothesize that CDs can influence the production of these two pigments too. Our aim was also to develop a small-volume high-throughput quick response test system for characterizing the influence of CDs on pyoverdine production. In addition, in the case of pyocyanin pigment quantification, we also investigated the possibility of eliminating the chloroform extraction step due to environmental and health issues.

## Materials and methods

### Applied bacterium strain and its cultivation

The bacterial strains *Pseudomonas aeruginosa* DSM (DSM 1117, ATCC 27853) and *Pseudomonas aeruginosa* PAO1 (DSM 22644, ATCC 15692) were cultured and maintained on agar slant cultures in the laboratory using LabM Luria-Bertani (LB) broth solidified with 2% agar. For the QS experiments 16-h-old (overnight) cell culture was prepared by inoculating 30 mL of LB broth with one loopful of bacterial colony shaken at 160 rpm and 30 °C.

The yield of pigment production both for pyoverdine and pyocyanin was studied under different growth conditions where the tested influential factors were the effect of temperature (30 °C and 37 °C), time (24, 48, 72 h), growth medium (King A, King B, LB, and LB + 2% glucose), agitation (no or 160 rpm) and test volume (200 μL or 30 mL).

### Applied cyclodextrin molecules

Since this is the first series of experiments systematically examining the impact of cyclodextrins (CDs) on the production of pyoverdine and pyocyanin by *P. aeruginosa*, our objective was to assess the effects of native CDs (ACD, BCD) and evaluate the impact of some frequently employed derivatives. The CDs employed in our experiments were all high-quality chemical products sourced from CycloLab Ltd. Key chemical properties, abbreviations, and the degree of substitution of the CDs under investigation are summarized in Table 1. For the average molecular structure of the tested cyclodextrins please refer to Fig. 6 in Berkl et al. (2022). To investigate concentration-dependent effects, the tested CD molecules were dissolved in sterile distilled water, and once complete dissolution was achieved, the stock solutions were adjusted to a concentration of 50 mM and subsequently sterile filtered through a 0.22-μm pore size filter. Since our main goal in the case of testing pyocyanin production was the development of an environmentally friendly and reliable method, we only studied the effect of two native CDs (ACD and BCD molecules without any substitution of side alkyl chains) at 1, 5, and 10 mM concentration levels. GCD did not prove to be effective in previous investigations (Molnár et al. 2021; Berkl et al. 2022).

**Table 1:** Main chemical properties and abbreviations of the tested α- and β-cyclodextrin parent molecules and their derivatives

α-cyclodextrins	A^1^	AMF^2^	MW^3^ [g/mol]	WS^4^ [g/L]	DS^5^
Native α-CD	ACD	C_36_H_60_O_30_	972	145	-
Randomly methylated α-CD	RAMEA	C_36_H_60_-nO_30_ (CH_3_)n	1127	> 500	11
Trimethyl-aminopropyl α-CD	QAACD	C_48_H_80_-nO_40_ (C_6_H_15_ONCl)n	1430	> 500	2.5–4
α-CD polymer	ACDPS	-	40,000*	> 500	-
β-cyclodextrins	A^1^	AMF^2^	MW^3^ [g/mol]	WS^4^ [g/L]	DS^5^
Native β-CD	BCD	C_42_H_70_O_35_	1135	18	-
Randomly methylated β-CD	RAMEB	C_42_H_70_-nO_35_ · (CH_3_)n	1303	> 500	12
Trimethyl-aminopropyl β-CD	QABCD	C_42_H_70_-nO_35_ (C_6_H_15_ONCl)n	1665	> 500	3–4
β-CD polymer	BCDPS	-	87,000**	> 500	-

*The molecular weight of a unit containing one CD molecule is 1390

**The molecular weight of a unit containing one CD molecule is 1620

^1^Abbreviation

^2^Average molecular formula; *n* = DS

^3^Molecular weight

^4^Water solubility at 25 °C

^5^Degree of substitution

The pyoverdine production of *P. aeruginosa* DSM 1117 was tested at 0.5, 2.5, and 12.5 mM CD concentrations in a small-volume (200 μL) model system applying 96-well microtiter plates.

### The quantification of pigment production

Preliminary experiments were carried out both for the pyoverdine and pyocyanin production applying the following excitation and emission wavelength pairs [400 ex; 480 em], [400 ex; 520 em], and [485 ex; 520 em] measuring a five-member twofold dilution series of cell suspensions containing pyoverdine or pyocyanin pigments. The optimal excitation wavelength and emission filter were aimed to be selected based on the values recommended in the literature (Meyer and Abdallah 1978; Albesa et al. 1985) using the available instrument, Fluostar Optima BMG Labtech microplate reader.

Regarding the pyocyanin pigment quantification, it was also verified, whether the presence of the cells in the cell suspensions disturbs the quantification of pigment molecules. For this reason, before and after centrifugation of the original cell suspension, we prepared the same dilution series with the appropriate growth medium.

The samples were centrifuged at 8500 rpm for 10 min, then 200-μL sample was pipetted into the wells of a 96-well microtiter plate in four parallels and the fluorescence was measured. A linear line was fitted onto each dataset in Microsoft Office Excel and the optimal wavelength and sample processing (with or without the separation of the cell mass and the supernatant) combination was selected based on the best linear fit (*R*^2^ value closest to 1).

In the case of pyocyanin quantification, the conventional chloroform extraction method was also compared to the method determining the amount of pyocyanin present in the original cell suspension (without extraction) and in the supernatant based on fluorescence intensity at 485 nm excitation and 520 nm emission wavelength with the Fluostar Optima BMG Labtech microplate reader in QUICK FITOP mode. In this experiment, a four-member dilution series of the supernatant containing pyocyanin was tested with and without chloroform extraction. A linear regression analysis was conducted on both datasets in Microsoft Office Excel and the performance of each method was assessed based on the best linear fit (*R*^2^ value closest to 1).

#### Conventional pyocyanin quantification with chloroform extraction

Three milliliters of chloroform was added to 5 mL supernatant and vortexed for 2 × 10 s. After 10 min of centrifugation at 8000 rpm at ambient temperature, pyocyanin could be seen in the lower phase in blue color. Two milliliters of the lower phase containing pyocyanin was pipetted into another centrifuge tube, then 1 mL of 0.2 n HCl was added and vortexed for 2 × 10 s. After 10 min of centrifugation at 8000 rpm at ambient temperature, ionized pyocyanin appears in the upper phase in pink color. The pyocyanin content of this upper phase was measured by applying an excitation wavelength of 485 nm and an emission wavelength of 520 nm with a Fluostar Optima BMG Labtech microplate reader after pipetting 3 × 300-μL sample into a 96-well microtiter plate. 0.2 n HCl solution was applied as a blank sample.

### Quorum sensing (QS) experimental setups

#### QS studies on pyocyanin production in a large-volume test system with *P. aeruginosa* DSM 1117

QS studies were performed in Erlenmeyer flasks (30 mL) freshly inoculated with *P. aeruginosa* DSM 1117 cell culture. The test started from the moment of inoculation in the presence of the administered dose of sterile CD solutions. In this series of experiments, an overnight cell culture of *P. aeruginosa* DSM 1117 (OD_600_ ≈ 1) was prepared and then diluted with 10× King A (King et al. 1954) medium for the experiments. In 100-mL volume Erlenmeyer flasks fresh King A medium (KA) with *P. aeruginosa* bacterial inoculant (PA) and different concentrations of CD solutions (CDS) were added using three parallels. The ratio of the KA:PA:CDS was 24:3:3 mL. The effect of ACD and BCD on the production of pyocyanin was tested in 1, 5, and 10 mM concentrations in large-volume test systems (30 mL). The Erlenmeyer flasks were shaken in an incubator set at 30 °C at 160 rpm in the dark for 72 h. Sterile distilled water was used as a control. At the end of the incubation period, the samples were centrifuged at 8500 rpm for 10 min in 50 mL volume conical tubes, then 3 × 300-μL supernatant sample was pipetted from each Erlenmeyer flask into a 96-well, round-bottomed Sarstedt microtiter plate in four replicates. Fluorescence intensity (FI) of the wells was determined using an excitation wavelength of 485 nm and an emission wavelength of 520 nm with Fluostar Optima BMG Labtech microplate reader in QUICK FITOP mode.

The raw data were subjected to corrections prior to evaluation: subtraction of the FI measured in a well containing 30 μL distilled water and 270 μL King A medium (blank sample), then the FI value of each well was divided by the optical density value of the same well.

#### QS studies on pyoverdine production in small-volume test system with *P. aeruginosa* PAO1

Overnight cell culture of *P. aeruginosa* PAO1 (OD_600_ ≈ 1) was prepared for the test using LB broth and then diluted 100x with King B (King et al. 1954) medium before the experiment. To study the time- and concentration-dependent effects, the tested CD molecules (ACD, RAMEA, QAACD, ACDPS, BCD, RAMEB, QABCD, BCDPS) were dissolved/suspended in distilled water.

Then the 50 mM concentration stock solutions/suspensions were further diluted to 2 and 10 mM concentrations. From each member of the dilution series, 50 μL was pipetted in 6 parallels into the wells of a sterile 96-well, round-bottomed Sarstedt microtiter plate in six replicates. Sterile distilled water was used as a control. From the diluted *P. aeruginosa* PAO1 cell suspension 150 μL was pipetted into each well, resulting in a 4-times dilution of the initial concentration of the CD solution in the wells resulting in a total of 200 μL test volume.

The microtiter plates were incubated for 24 and 48 h at 37 °C in a thermostatic chamber. After 24 and 48 h of incubation, the fluorescence intensity (FI) of the wells was determined using an excitation wavelength of 485 nm and an emission wavelength of 520 nm with Fluostar Optima BMG Labtech microplate reader in QUICK FI TOP mode. All experiments were repeated three times. The raw data were subjected to corrections before evaluation: subtraction of the FI measured in a well containing 50 μL distilled water and 150 μL King B medium (blank sample). Specific fluorescence intensity (normalized to cell densities) was determined by dividing these values with the optical density measured at 630 nm. Applying this normalization with the optical density at 630 nm, we aimed to avoid the effect of growth rate.

### Cell growth quantification methods

To assess whether the cyclodextrins (CDs) exhibited any cytotoxic effects, we monitored bacterial population growth by measuring the optical density of the test medium (OD). In the case of the small-volume pyoverdine pigment production assay, cell growth was followed by determining the optical density (OD) at a wavelength of 630 nm with DIALAB ELx800 ELISA Microplate Reader (Dialab GmbH, Austria).

In the case of the large-volume pyocyanin pigment production assay, 200-μL sample was taken in three parallels of the test systems, then OD was determined at a wavelength of 485 nm with Fluostar Optima BMG Labtech microplate reader in QUICK ABS mode. After characterizing cell growth based on these data, we calculated the relative pigment production inhibition normalized to optical density for both pyoverdine and pyocyanin production.

### Statistical analysis

To evaluate the results of pyocyanin production, we conducted a one-way analysis of variance (ANOVA) using TIBCO Statistica™ 13.5 software (TIBCO Software, Inc., Palo Alto, CA, USA). This analysis aimed to identify statistically significant effects, with a significance level set at *p* < 0.05. We also conducted Univariate Tests of Significance and examined the homogeneity of variances. Regarding the evaluation of pyoverdine production, we employed repeated measures analysis of variance (RM ANOVA) using the same TIBCO Statistica™ 13.5 software. This analysis was conducted to investigate the potential influence of cyclodextrin concentrations, exposure time (incubation time), and their interactions on the pyoverdine production by *P. aeruginosa*, aiming to study whether the cyclodextrin treatments had significant effect at *p* < 0.05 significance level. For this purpose, the Newman-Keuls post hoc test was applied. The Mauchley sphericity test was utilized to confirm relevant criteria. Significant differences were indicated with distinct letters on columns on the diagrams. Please note that in all figures and tables, statistical significance between treatments is marked by lowercase letters in alphabetical order, where “a” is the smallest value. Values signed with the same letter indicate that there was no significant difference between them.

## Results

### Results of *P. aeruginosa* growth studies and pigment production

In the initial step of our research, we conducted preliminary experiments to characterize the growth of the *P. aeruginosa* bacterial strains (DSM 1117 and PAO1) under various conditions. The aim was to identify the optimal circumstances for achieving a high and selective yield of pigment production in the shortest possible cultivation period. The effect of temperature (30 °C and 37 °C), time (24, 48, 72 h), growth medium (King A, King B, LB, and LB + 2% glucose), agitation (no or 160 rpm), and test volume (200 μL or 30 mL) were tested both for pyoverdine and pyocyanin production.

Although the results of the preliminary experiments are not presented in detail, we opted to employ DSM 1117 *P. aeruginosa* bacterial strain for pyoverdine production assays due to the superior capability exhibited by this strain in pyoverdine production. Conversely, in the context of pyocyanin production, PAO1 strain demonstrated greater feasibility.

In the case of pyoverdine pigment production, an adequate yield could be reached in a small test volume (200 μL) without agitation after 24 h. The King B growth medium resulted in the best selectivity of pyoverdine production, and the higher temperature (37 °C) also had favorable effect on the pyoverdine yield.

In the case of pyocyanin pigment production in a small-volume (200 μL) test system with or without agitation, the bacterial strain did not produce pyocyanin, therefore the small-volume test system proved to be a dead end in this case even after 72 h of cultivation.

In large-volume test systems (30 mL) the King A medium resulted in the best selectivity of pyocyanin production. Temperature did not have a significant effect on pyocyanin yield, which became satisfactory after 72 h, at 160 rpm shaking, at 30 °C.

### Studies of quantification methods of pyoverdine and pyocyanin pigments—preliminary experiments

**Fig. 1:**
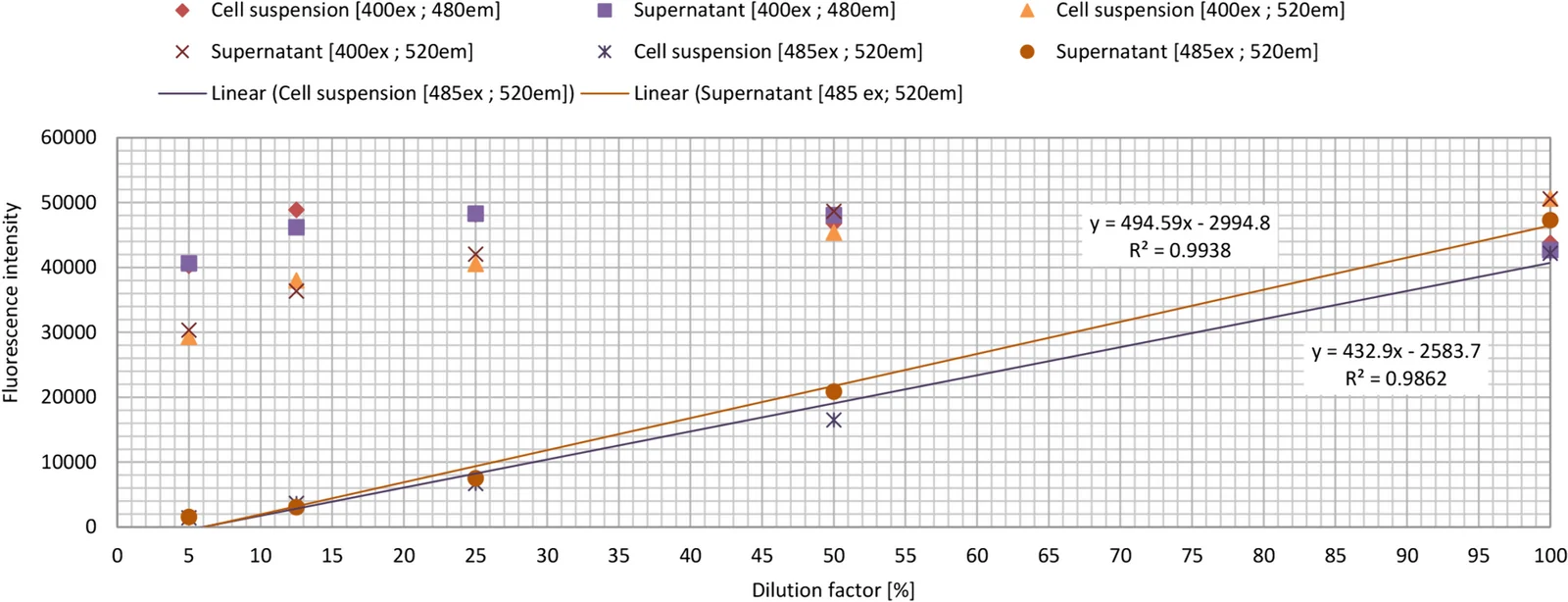
Fluorescence intensity of the *P. aeruginosa* PAO1 cell suspension and its supernatant containing pyoverdine measured at different excitation and emission wavelength combinations

In a series of preliminary experiments, the pyoverdine containing cell suspension and its supernatant were diluted with King B medium to reach 5, 12.5, 25, and 50% of the original pigment content. FI was determined at different excitation and emission wavelength pairs of [400 ex; 480 em], [400 ex; 520 em], and [485 ex; 520 em] nm. A linear trend line could not be fitted onto the data points of the measurements carried out with the wavelength pairs of [400 ex; 480 em] and [400 ex; 520 em] nm. Based on the *R*^2^ values of the linear fitting formula of the whole cell suspension and the supernatant analyzed at 485 ex and 520 em nm wavelengths, the presence of the cells in the cell suspensions did not disturb the quantification of pigment molecules; however, the *R*^2^ value of the trend line fitted to the data points belonging to the cell-free supernatant was closer to 1 (*R*^2^ = 0.9938) (Fig. 1).

The aforementioned procedure was carried out in the case of pyocyanin pigment determination in the whole cell suspension and the supernatant of *P. aeruginosa* cultivated and diluted in King A medium. A linear trend line could not be fitted to the data points measured with the wavelength pairs [400 ex; 480 em] and [400 ex; 520 em] nm.

**Fig. 2:**
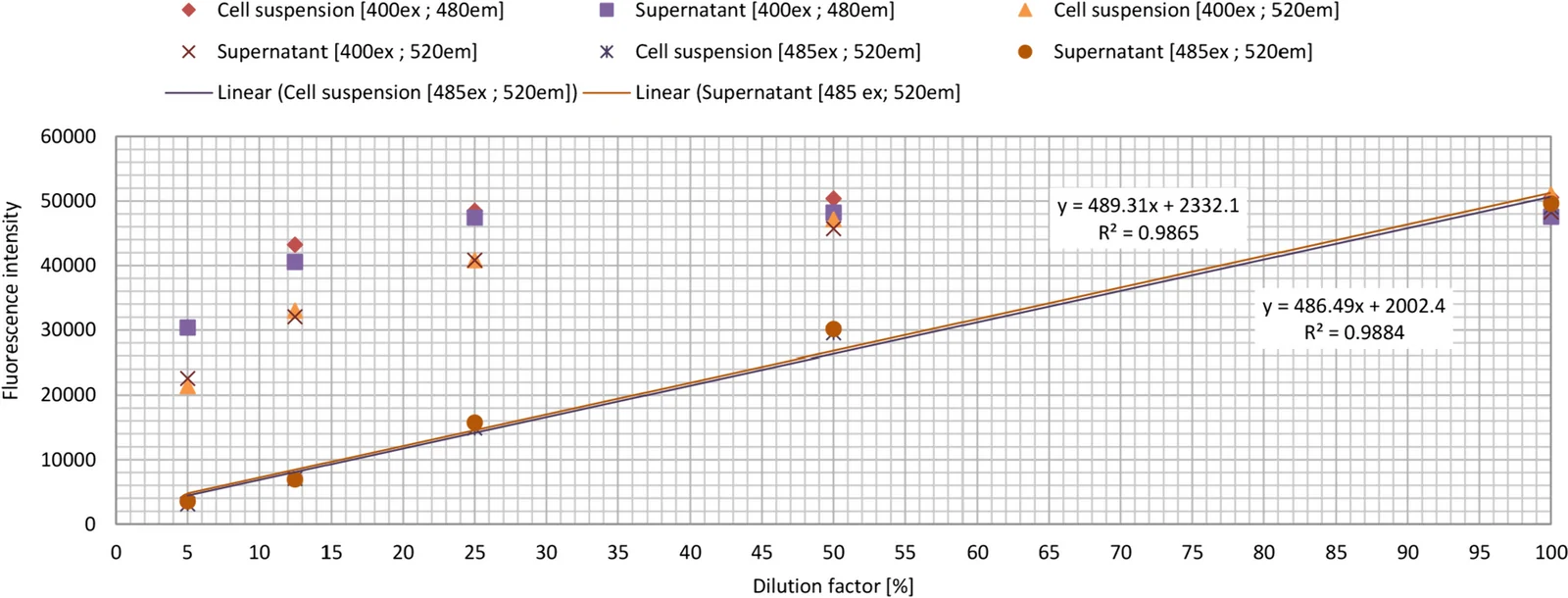
Fluorescence intensity of the *P. aeruginosa* DSM 1117 cell suspension and its supernatant containing pyocyanin measured at different excitation and emission wavelength combinations

The *R*^2^ values of the linear fitting formula of the whole cell suspension and the supernatant were analyzed at 485 ex and 520 em nm wavelengths. The results showed that the presence of the cells in the cell suspensions did not disturb the quantification of pigment molecules. The *R*^2^ values in both cases, i.e., cell suspension and supernatant, were very close to 1 (*R*^2^ = 0.9884 and 0.9865, respectively), as depicted in Fig. 2.

It was tested whether different CD molecules could complex the pigment molecules, namely pyoverdine, and pyocyanin, in the growth media used. However, no significant changes or tendencies in FI were observed when the CD molecules were added to the pigment-containing growth media in eight different concentrations (ranging from 0.049 to 12.5 mM) (data not shown).

**Fig. 3:**
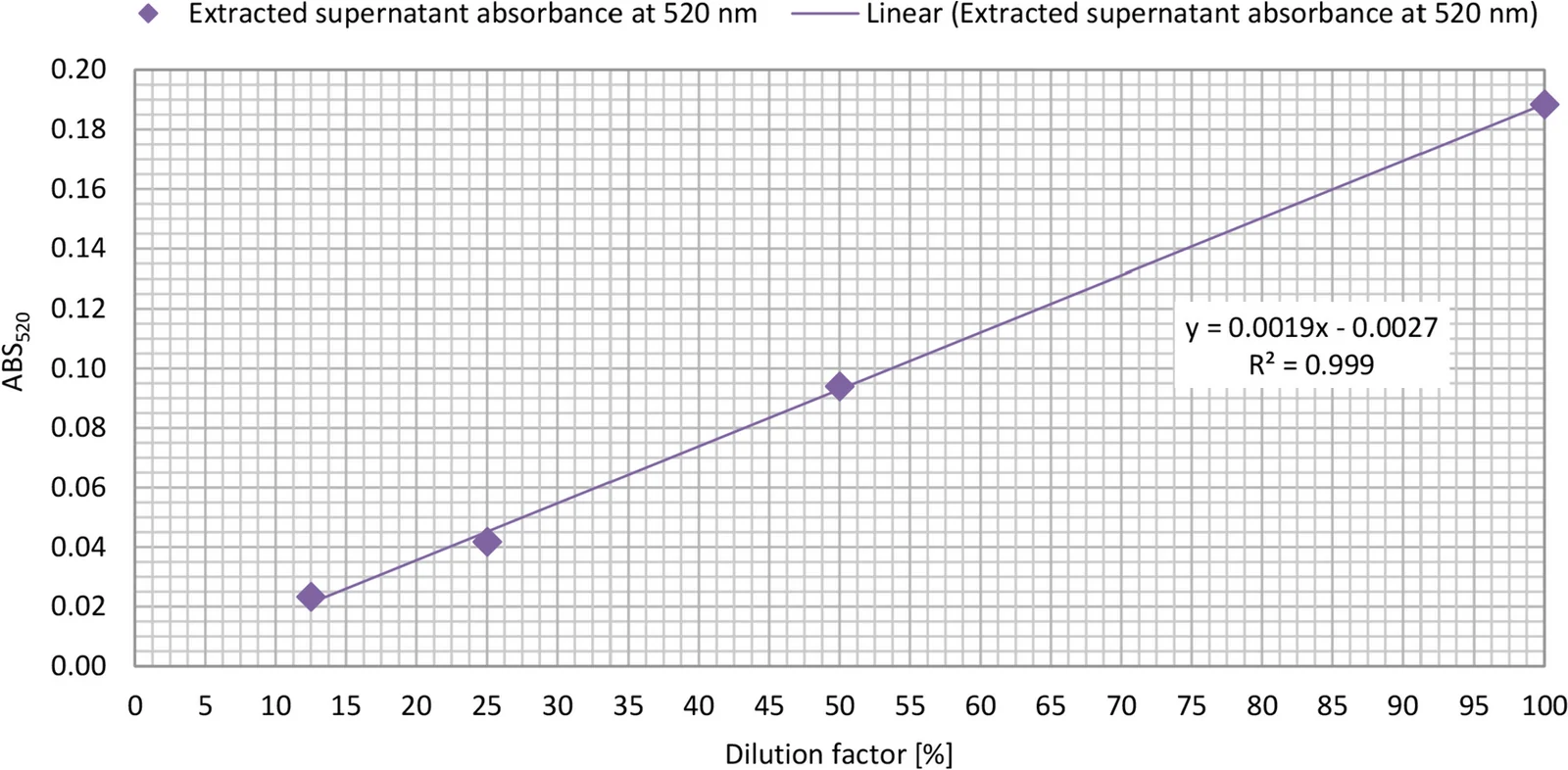
Absorbance of the chloroform extracted *P. aeruginosa* DSM 1117 supernatant containing pyocyanin at 520 nm

In the pyocyanin pigment quantification study, two methods were compared: the conventional chloroform extraction method and the method that determines the amount of pyocyanin in the original supernatant through fluorescence intensity at an excitation wavelength of 485 nm and an emission wavelength of 520 nm. Figure 3 displays the linear trend line fitted to the data points of the conventional chloroform extraction method. By comparing the *R*^2^ value (0.999) of this method to the *R*^2^ value of the linear fitting formula of the supernatant analyzed at 485 ex and 520 em nm wavelengths (Fig. 2), it can be concluded that the non-chloroform extraction method has a very good performance (*R*^2^ = 0.9884). Additionally, this method eliminates the environmental and health concerns attributed to the use of chloroform.

### Effect of cyclodextrins on pyocyanin pigment production of *P. aeruginosa* DSM 1117 in a large-volume test system

**Fig. 4:**
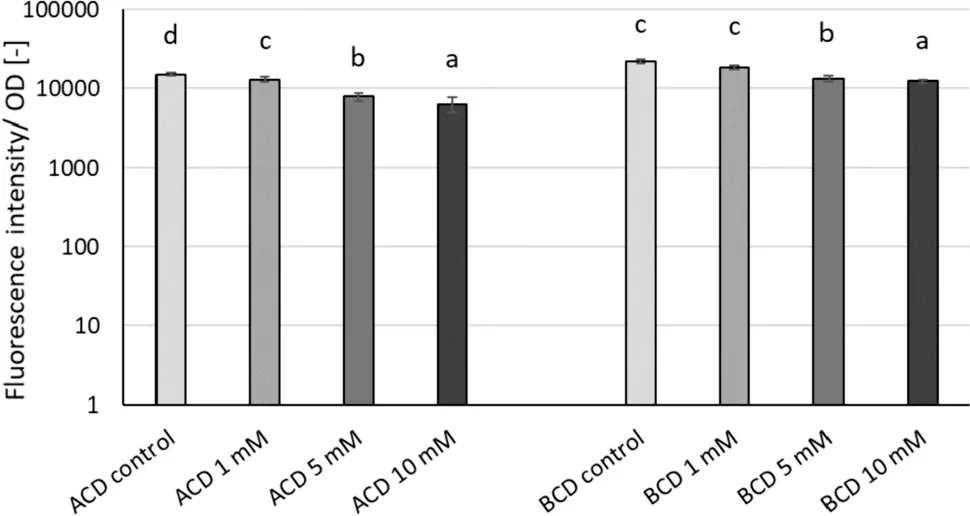
Modulation of relative pyocyanin production normalized to cell density by ACD and BCD parent CD molecules in the *P. aeruginosa* DSM 1117 large-volume model system. Statistical significance (*p* < 0.05) is marked by lowercase letters, where *a* indicates the smallest value. Values signed with the same letter indicate that there was no significant difference between them. Statistical analysis was carried out distinctively for ACD and BCD datasets. Data represent averages of three replicates

In the large-volume test system, both the administration of ACD and BCD significantly inhibited the production of pyocyanin. The inhibition percentage ranged from 36 to 58% at 5 and 10 mM concentrations (Fig. 4). However, at 1 mM concentration, BCD did not show any significant effect on pyocyanin production, while 1 mM of ACD significantly inhibited pyocyanin production by 13%.

Table 2 shows the relative cell growth compared to control in the case of each tested concentrations of ACD and BCD based on OD measurements (Supplementary Tables S1), while Table 3 shows the inhibition of pyocyanin pigment production compared to control and normalized to cell growth (OD).

**Table 2:** Inhibition of cell growth [%] compared to control in the tested ACD and BCD concentrations in the *P. aeruginosa* DSM 1117 large-volume model system. Significant inhibition or stimulation compared to control is marked by bold italics

Concentration [mM]	ACD	BCD
1	1.5 ± 2.3	***−***3.5 ± 2.9
5	***−17.1 ± 1.4***	***−11.4 ± 2.5***
10	***−28.3 ± 4.0***	0.8 ± 3.6

**Table 3:** Inhibition of pyocyanin pigment production [%] compared to control in the tested ACD and BCD concentrations in the *P. aeruginosa* DSM 1117 large-volume model system. Statistical significance (*p* < 0.05) is marked by lowercase letters, where *a* indicates the smallest value. Values signed with the same letter indicate that there was no significant difference between them

Concentration [mM]	ACD	BCD
1	13 ± 7 a	12 ± 5 a
5	47 ± 6 b	36 ± 6 b
10	58 ± 9 c	41 ± 3 c

As illustrated by the results, we did not observe any inhibitory effect on population growth. Instead, we observed a stimulating effect in terms of relative cell growth compared to the control. It has to be noted, that despite this significant positive effect on cell growth, the production of pyocyanin was reduced by ~60%.

### Effect of cyclodextrins on pyoverdine pigment production of *P. aeruginosa* PAO1 in a small-volume test system

The potential quorum quenching effect of α- and β-CD (ACD, BCD), randomly-methylated β-CD (RAMEB), quaternary ammonium α- and β-CD (QAACD, QABCD), (2-hydroxypropyl)-α- and β-CD (HPACD, HPBCD), sulfobutyl ether β-CD (SBEBCD), and α- and β-CD polymers (ACDPS, BCDPS) on the pyoverdine production of *P. aeruginosa* was tested at 0.5, 2.5, and 12.5 mM CD concentrations in a small-volume (200 μL) model system. The choice of these derivatives was based on our previous experience in the biofilm formation studies of *P. aeruginosa* PAO1 (Berkl et al. 2022).

**Fig. 5:**
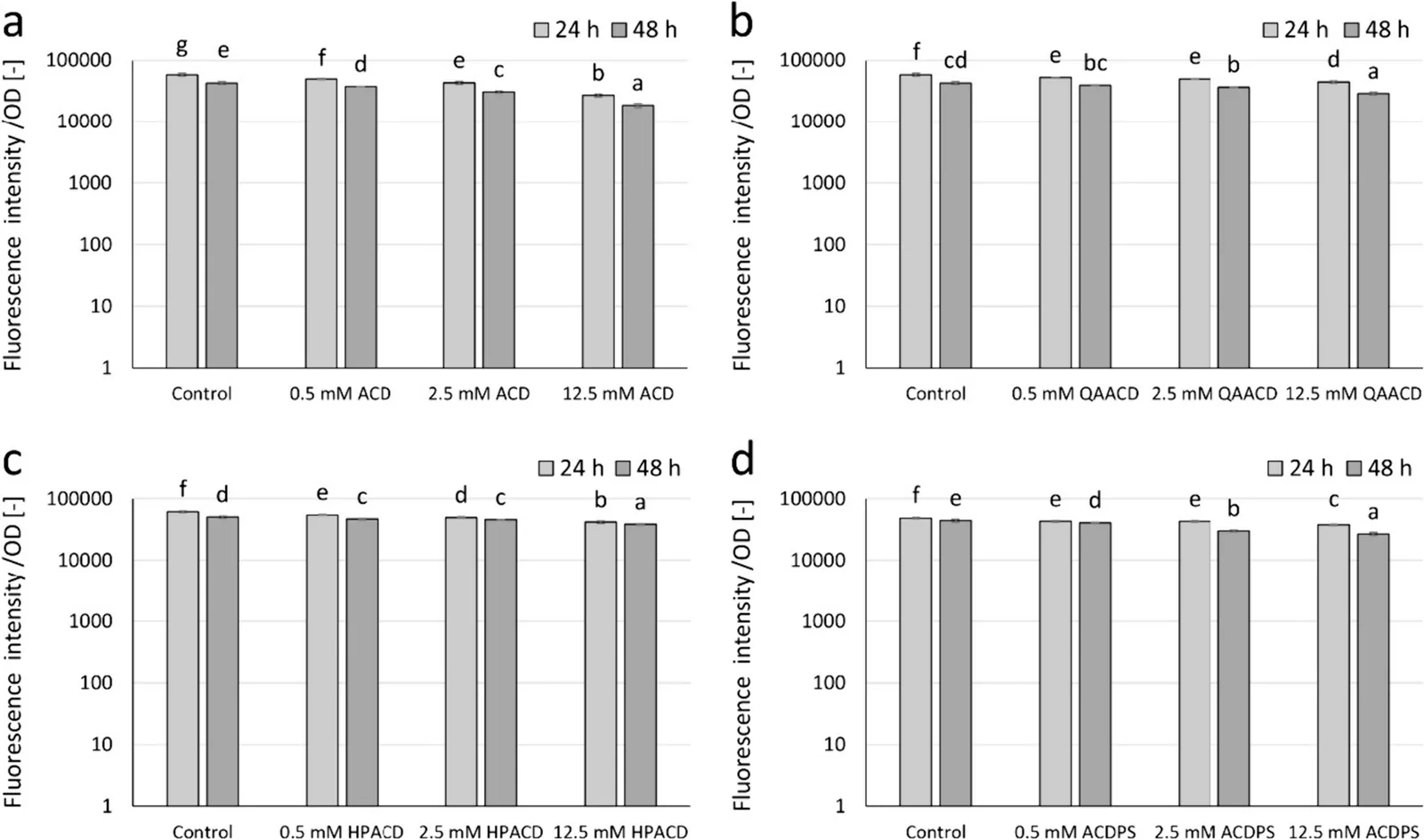
Effect of increasing concentrations of ACD (**a**), QAACD (**b**), HPACD (**c**), and ACDPS (**d**) on pyoverdine pigment production of *P. aeruginosa* PAO1 after 24- and 48-h exposure time normalized to cell density. Statistical significance (*p* < 0.05) is marked by lowercase letters, where *a* indicates the smallest value. Values signed with the same letter indicate that there was no significant difference between them. Data represent averages of five replicates

Significant inhibition of pyoverdine pigment production was observed with native ACD and its tested derivatives, with inhibition ranging from 12 to 57% depending on exposure time, type, and concentration of the CD. As illustrated by Fig. 5, the extent of inhibition was significantly influenced by the concentration of CD. Generally, the inhibition was greater at the higher concentrations. After 24 h of exposure, the highest concentration (12.5 mM) of ACD, QAACD, HPACD, and ACDPS resulted in an inhibition rate of 54%, 24%, 32%, and 23%, respectively. While after 48 h of exposure, the inhibitory effect was found to be 57%, 32%, 24%, and 49%, respectively, for ACD, QAACD, HPACD, and ACDPS (Fig. 5).

According to the results obtained by testing the highest applied concentrations (12.5 mM) of ACD and its derivatives, it was found that ACD was the most effective in reducing pyoverdine production. The decrease was significant after 24 and 48 h of exposure with inhibition percentages of 54 and 57%, respectively. After 48-h exposure to 12.5 mM concentration, the derivatives of ACD showed 24–39% inhibition in the following order of efficiency: ACDPS > QAACD > HPACD.

The results of the repeated measures variance analyses (RMANOVA) conducted on ACD and ACD derivatives showed that ACDs were effective as shown in Table 4. Both the duration of contact and the treatments with CD had a significant impact on the bacteria’s ability to produce pyoverdine pigment.

**Table 4:** RMANOVA results over time to evaluate the effect of ACD and ACD derivatives on the pyoverdine pigment production of *P aeruginosa* PAO1. Bold numbers indicate significant differences at *p* < 0.05

Source of variation	Df^1^	MS^2^	*F*^3^	*p*^4^
ACD				
ACD treatment	**3**	**1.469E+09**	**198.68**	**0.000**
Time	**1**	**1.321E+09**	**910.99**	**0.000**
Time × ACD treatment	**3**	**2.153E+07**	**14.85**	**0.000**
QAACD				
QAACD treatment	**3**	**3.310E+08**	**50.93**	**0.000**
Time	**1**	**1.465E+09**	**1373.93**	**0.000**
Time × QAACD treatment	3	1.923E+06	2.74	0.000
HPACD				
HPACD treatment	**3**	**4.805E+08**	**115.86**	**0.000**
Time	**1**	**4.249E+08**	**625.29**	**0.000**
Time × HPACD treatment	**3**	**3.452E+07**	**50.79**	**0.000**
ACDPS				
ACDPS treatment	**3**	**4.066E+08**	**171.69**	**0.000**
Time	**1**	**5.879E+08**	**306.51**	**0.000**
Time × ACDPS treatment	**3**	**6.385E+07**	**33.29**	**0.000**

^1^Degree of freedom

^2^Mean square

^3^*F*-ratio

^4^*p*-value

**Fig. 6:**
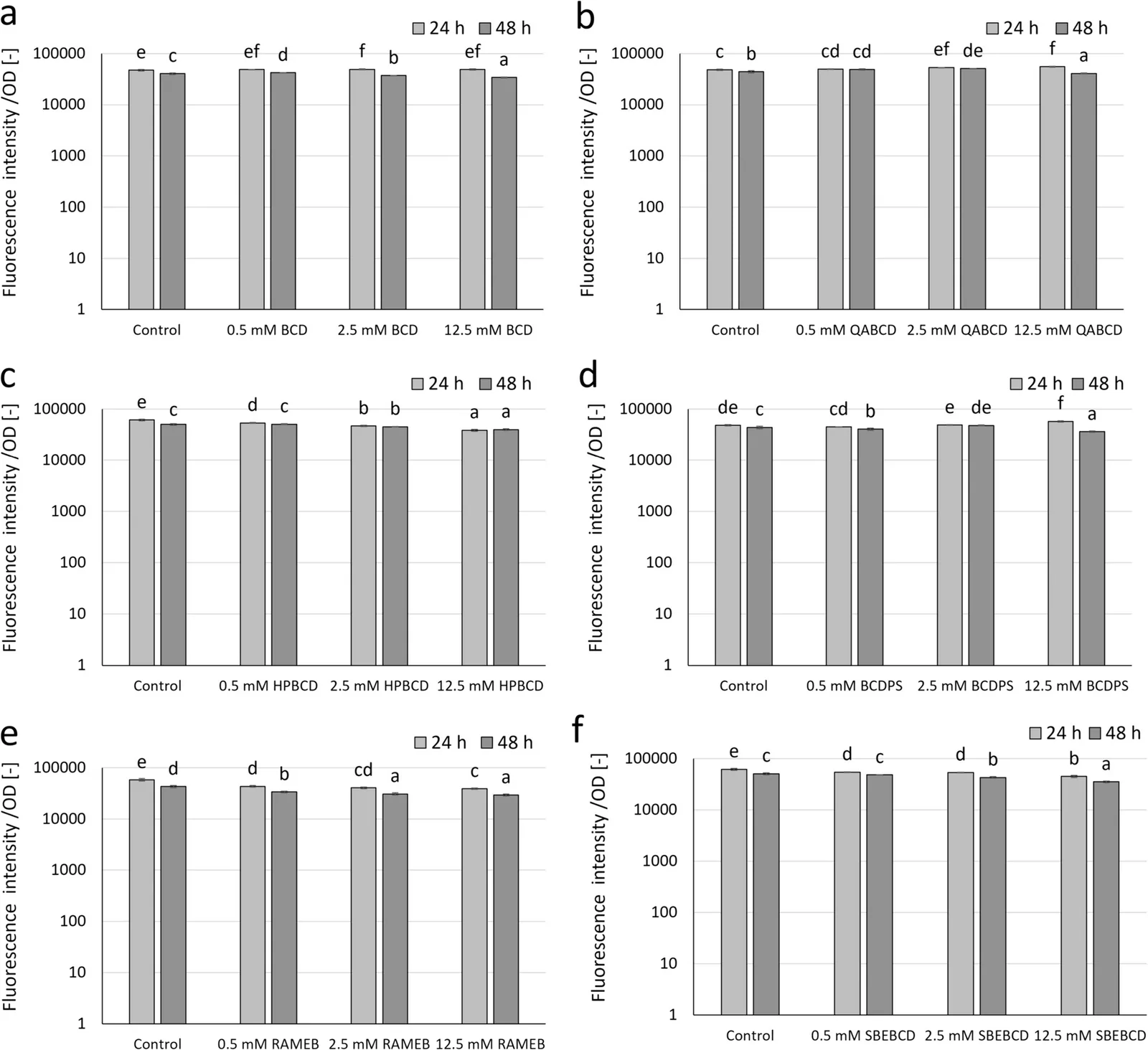
Effect of increasing concentrations of BCD (**a**), QABCD (**b**), HPBCD (**c**), BCDPS (**d**), RAMEB (**e**), and SBEBCD (**f**) on pyoverdine pigment production of *P. aeruginosa* PAO1 after 24- and 48-h exposure time normalized to cell density. Statistical significance (*p* < 0.05) is marked by lowercase letters, where *a* indicates the smallest value. Values signed with the same letter indicate that there was no significant difference between them. Data represent averages of five replicates

Figure 6 shows the potential quorum quenching effect of β-CD (BCD) and its derivatives on the pyoverdine production of *P. aeruginosa* PAO1 at concentrations of 0.5, 2.5, and 12.5 mM. The modulation of pyoverdine production by BCD and its derivatives, particularly BCD, QABCD, and BCDPS seemed to be different from ACD and its derivatives.

HPBCD, RAMEB, and SBEBCD significantly decreased pyoverdine production in the applied concentrations after 24-h exposure showing a concentration-dependent trend. On the other hand, BCD, QABCD, and BCDPS were not effective in inhibiting pyoverdine production. For these CDs (BCD, QABCD, and BCDPS), a significant inhibitory effect was observed only after 48 h.

After 48-h exposure to the highest concentration (12.5 mM), the inhibition of pyoverdine pigment production ranged from 7 to 31% for BCD and its derivatives.

The results of repeated measures variance analyses (RMANOVA) of BCD and BCD derivatives (Table 5) demonstrated that the BCDs were significantly efficient; both the contact time and the CD treatments have influenced the pyoverdine pigment production capacity of the bacteria.

**Table 5:** RMANOVA results over time to evaluate the effects of BCD and BCD derivatives on the pyoverdine pigment production of *P. aeruginosa* PAO1. Bold numbers indicate significant differences at *p* < 0.05

Source of variation	Df^1^	MS^2^	*F*^3^	*p*^4^
BCD				
BCD treatment	**3**	**1.993E+07**	**22.43**	**0.000**
Time	**1**	**7.536E+08**	**406.16**	**0.000**
Time × BCD treatment	**3**	**3.265E+07**	**17.60**	**0.000**
QABCD				
QABCD treatment	**3**	**4.472E+07**	**17.49**	**0.000**
Time	**1**	**2.139E+08**	**133.26**	**0.000**
Time × QABCD treatment	**3**	**6.042E+07**	**37.64**	**0.000**
HPBCD				
HPBCD treatment	**3**	**5.661E+08**	**152.04**	**0.000**
Time	**1**	**1.192E+08**	**76.79**	**0.000**
Time × HPBCD treatment	**3**	**7.406E+07**	**47.72**	**0.000**
BCDPS				
BCDPS treatment	**3**	**3.055E+07**	**9.73**	**0.001**
Time	**1**	**5.176E+08**	**170.81**	**0.000**
Time × BCDPS treatment	**3**	**1.500E+08**	**49.48**	**0.000**
RAMEB				
RAMEB treatment	**3**	**5.863E+08**	**98.77**	**0.000**
Time	**1**	**1.171E+09**	**686.60**	**0.000**
Time × RAMEB treatment	**3**	**2.046E+07**	**12.00**	**0.000**
SBEBCD				
SBEBCD treatment	**3**	**4.967E+08**	**134.99**	**0.000**
Time	**1**	**8.657E+08**	**422.87**	**0.000**
Time × SBEBCD treatment	**3**	**1.154E+07**	**5.64**	**0.008**

^1^Degree of freedom

^2^Mean square

^3^*F*-ratio

^4^*p*-value

In the small-volume pyoverdine pigment production test system, cell growth was also monitored throughout the series of experiments. Based on optical density measurement (Supplementary Table S2), the cell growth inhibition values did not show a significant decrease exceeding 20% triggered by CDs compared to the control. However, 12.5 mM HPBCD resulted in a 22.5 ± 0.6% decrease in population growth after 24 h. At the same time, significant stimulation was observed in some cases. For BCD, QABCD, ACDPS, and BCDPS cell growth was significantly stimulated at some concentrations.

## Discussion

### Effect of cyclodextrins on pyocyanin pigment production of *P. aeruginosa* DSM 1117

As inhibiting pyocyanin production is a promising antivirulence approach for managing *P. aeruginosa* infections, researchers investigated the potential QQ effect of the antipseudomonal molecules of different structure (Kalia 2013; El-Shaer et al. 2016; Ugurlu et al. 2016; Kalia et al. 2018; Rubini et al. 2019; Abbas et al. 2020; Ramírez-Rueda and Salvador 2020; Dong et al*.* 2001; Maisetta et al. 2021).

The objective of this study was twofold: first, to examine the time- and concentration-dependent QQ effect of α- and β-CD molecules and their derivatives on the pyoverdine and pyocyanin pigment production in the *P. aeruginosa* model system without directly affecting bacterial viability. Secondly, our research has focused on comparing the efficacy of cyclodextrin and other agents in influencing QS-driven processes.

In a study conducted by Ugurlu et al. (2016), the effects of plant-derived phenolic compounds on the production of pyocyanin in *P. aeruginosa* were investigated using the chloroform extraction method. The results showed a decrease in pyocyanin production by 9–21% when 4 mmol/L of vanillic acid, caffeic acid, cinnamic acid, and ferulic acid were present, compared to control. Ahmad et al. (2015) found that plant volatiles, such as linalool, thujone, and citral inhibited pyocyanin production by ~55–75%, at 0.125–0.5 mg/ml concentrations. On the other hand, Maisetta et al. (2021) used a low-molecular-weight quaternized chitosan derivative (QAL) to reduce pyocyanin levels in the supernatants of *P. aeruginosa* ATCC, Pa W4, and Pa B910. They observed that exposure to 0.62 mg/mL QAL resulted in a reduction of pyocyanin levels by 60, 85, and 56%, in each of the three strains, respectively.

Nanomaterials have attracted great attention recently due to their potential QQ effect in *P. aeruginosa* (Huh and Kwon 2011; Gómez-Gómez et al. 2019; Hayat et al. 2019; Khan et al. 2020). Jabłońska et al. (2022) assessed modulated pyocyanin production by multi-walled carbon nanotubes and nano zinc oxide using the chloroform–hydrochloric acid method. The study found that high concentrations (500 μg/mL) of multi-walled carbon nanotubes had a stimulatory effect on pyocyanin production, while lower dosages (125 μg/mL) augmented pyocyanin production. On the other hand, high concentrations (500 μg/mL) of nano zinc oxide inhibited pyocyanin production by around 90%, while the lower concentration (7.31 μg/mL) led to an increase in pigment production.

Our study involved a large-volume test system (30 mL) that showed 58% inhibition of pyocyanin production with exponentially growing bacterial cultures. This was achieved after 72-h incubation by applying 10 mM ACD without negatively affecting cell growth and viability. In addition, the 10 mM ACD concentration had a statistically significant, 28% stimulating effect in terms of relative cell growth compared to the control. It is important to note, that we achieved a 58% inhibition of pyocyanin production while also stimulating cell growth. We also found that we could simplify the conventional method of using chloroform to eliminate the chloroform extraction step of pyocyanin pigment molecules. This resulted in a more environmentally- and health-friendly method. Our study found that α- and β-CD molecules are as effective as several agents reported in the current literature for inhibiting pyocyanin pigment production. Therefore, α- and β-CD molecules could be representatives of a novel structural sub-class of pyocyanin inhibitors, with potential for exploitation in a therapeutic context, contributing to the development of novel antipseudomonal agents. Furthermore, we demonstrated that both pyocyanin production and the effect of CDs could be reliably monitored in the original test system without using different extraction procedures.

### Effect of cyclodextrins on pyoverdine pigment production of *P. aeruginosa* PAO1

The production of yellow-green, water-soluble, fluorescent pyoverdine pigment by *P. aeruginosa* is one of the QS-controlled factors of this Gram-negative bacterium and is presumably regulated by both AHL- and AHQ-based systems (Stintzi et al. 1998; Diggle et al. 2007; Li et al. 2007; Buzid et al. 2016). This Gram-negative bacterium synthesizes the pigment mainly under iron deficiency conditions (Meyer 2000; Cézard et al. 2015) to capture iron and cause acute infections (Cézard et al. 2015). The production of pyoverdine pigment is a quorum-sensing dependent process and the pyoverdine has the ability to regulate its own production (Cézard et al. 2015). This is required to cause infection through biofilm formation. Therefore, it is crucial to develop effective methodologies to decrease the production of this pigment.

Our research aimed to evaluate the effect of CDs on the pyoverdine production capacity of *P. aeruginosa*, over time and concentration, as well as to develop and test a small-volume microtiter plate test system that can sensitively and reliably monitor pyoverdine production, routinely. As far as we know, there is no established high-throughput monitoring system that can quantify pyoverdine production.

However, to demonstrate the applicability of a new QS inhibitor we need to take a systematic approach and establish high-throughput procedures to develop a unique methodology.

Although it has been demonstrated that pyoverdine production plays an important role in biofilm formation and pathogenicity (Girard and Bloemberg 2008), the QQ effect of molecules with potential modulatory effect on pyoverdine pigment production is barely investigated (Sepahi et al. 2015; Ramírez-Rueda and Salvador 2020), and even less is known about the efficiency of CD molecules.

It has been observed that various factors such as the dimensions of the CD cavity, the configuration of substituent groups, the monomeric or polymeric character of the CD, and the concentration of these cyclic oligosaccharides collectively affect the production of pyoverdine pigment. Previous studies have already shown that CDs have the potential to act as quorum quenchers (QQ) in other model systems and with different endpoints (Kato et al. 2006; Kato et al. 2007; Morohoshi et al. 2013). However, native CDs were generally less effective in these studies than CD derivatives. Regarding our research results, the β-CD derivatives were more effective than native BCD, while among the α-CDs, the native ACD showed the highest efficiency. This may be due to differences in test organisms and signal molecules. However, given the complexity of these systems, other factors and their interactions may also affect the outcomes. Therefore, it is essential to perform individual “CD by CD” studies for each endpoint (virulence factor) of each bacterium to draw reliable conclusions about the effectiveness of CDs.

Several studies have been conducted on the production of siderophores in *Pseudomonas* species, and it has been observed that the regulation of siderophore production is a complex process. Additionally, it has been found that various factors, such as the pH and the composition of the media including the availability of iron, sulfur, and phosphate, and nitrogen sources can influence this process. For example, pyoverdine production is affected by these factors (Albesa et al. 1985; Ringel and Brüser 2018; Vindeirinho and Soares 2021). In our e experiments, we used the same culture medium throughout and the pH remained constant. Therefore, any observed effects can be attributed to the presence of cyclodextrins. Nevertheless, it is important to investigate the influence of CDs on nutrient availability in future studies.

According to our previous studies (Berkl et al. 2022), the autoinducer-dependent quorum sensing mechanism of biofilm formation in *Pseudomonas aeruginosa* model system was significantly inhibited by cyclodextrins. The quorum quenching effect (decreased biofilm formation capacity) of these cyclic oligosaccharides was clearly demonstrated. Berkl et al. (2022) found that among all the tested CDs, ACD was the most efficient in reducing the process regulated by QS. This points to a connection between biofilm formation and pyoverdine production, which has already been confirmed by other researchers (Kang et al. 2017; Díaz-Pérez et al. 2022). These studies indicate that biofilm formation contributes to the virulence of pathogenic microorganisms by regulating the production of the pyoverdine siderophore. The effectiveness of ACD is likely due to its small cavity size among CDs, which enables it to form a more stable complex with the acyl chain of the signals (Molnár et al. 2021; Berkl et al. 2022; Poulson et al. 2022). Among β-cyclodextrins, RAMEB was generally the most effective in both biofilm development and pyoverdine production, possibly because of its higher water solubility (Jansook et al. 2018; Poulson et al. 2022). The water solubility of β-CD is lower (18.5 mg/mL, 25 °C) than that of native α-CD (130 mg/mL, 25 °C), but it can be greatly increased by methylation in the case of RAMEB (> 600 mg/mL, 25 °C). The available space in the CD cavity and the water solubility both play an important role in the QQ efficiency.

It is worth noting that the derivatization of natural CDs can negatively affect their QQ efficiency. This is demonstrated by the inhibitory effect of quaternary amino derivatives on biofilm development (Berkl et al. 2022), pyoverdine production (present study), and our previous QS research on the *A. fischeri* model system (Molnár et al. 2021).

Our study has shown that the microtiter plate assay is an effective high-throughput screening tool for assessing the impact of cyclodextrins (CDs) on the fluorescent pigment production capability of *P. aeruginosa*. Previously, research has relied on a qualitative plate method of Ramírez-Rueda and Salvador (2020) which used a visual reading at 366 nm wavelength to record the presence or absence of the emitted fluorescence by the *P. aeruginosa* spot in the plate. Positive results were recorded when fluorescence was not observed and negative results were recorded when any amount of fluorescence was observed. Our study demonstrated that the high-throughput microtiter plate test system was a reliable, quantitative method for the time- and concentration-dependent modulation of pyoverdine pigment production.

Our study concluded that CDs have a significant impact on the production of pyocyanin and pyoverdine. As expected, different CDs with different structures had varying effects on pyoverdine production. The native ACD showed the highest attenuation activity in pyoverdine production. Based on our results, ACD was more effective than its derivatives and β-CDs. When we compared our experiments on pyoverdine production with our previous research on biofilm formation, we found that the regulation of the siderophore pyoverdine production could be linked to biofilm formation, which contributes to pathogen virulence. Our results confirm the great potential of CDs as antivirulence mediators, even though the available scientific literature does not show any references about the anti-pyoverdine activity of CDs. Our research also demonstrated a quantitative method to characterize QS-mediated pyocyanin production in *P. aeruginosa* for the detection of the effects of cyclodextrins.

Although the modulatory effect of CDs on QS-driven processes has been demonstrated, there are still further/unanswered questions. In future research, we plan to investigate the impact of adding signal molecules to pyocyanin and pyoverdine pigment production in combination with CDs. This approach has previously proven effective in our studies (Molnár et al. 2021).

We also aim to determine the complex association constants of the signalling molecules with the selected CDs. Our long-term goals are to explore, how different CD molecules, including more than one signal molecule can affect QS-controlled processes in *P. aeruginosa.* Additionally, we will assess the effects of CD structures on these processes.

## Supplementary information

ESM 1 (PDF 242 kb)

## Data Availability

Experimental data are available within this research article and in the related Supplementary Materials. The raw datasets generated during and/or analyzed during the current study are available from the corresponding author on reasonable request.
