# Supplementary material for: Quorum quenching effect of cyclodextrins on the pyocyanin and pyoverdine production of Pseudomonas aeruginosa
Source: Appl Microbiol Biotechnol. 2024 Mar 22;108:271. doi: 10.1007/s00253-024-13104-7 (PMC10959793; doi:10.1007/s00253-024-13104-7)
Supplement: Supplementary file 1 [file 253_2024_13104_MOESM1_ESM.pdf]

SUPPLEMENTARY MATERIAL

Quorum quenching effect of cyclodextrins on the pyocyanin and pyoverdine production of *Pseudomonas aeruginosa*

Ildikó Fekete-Kertész[1], Zsófia Berkl[1], Kata Buda[1], Éva Fenyvesi[2], Lajos Szente[2], Mónika Molnár[1]

1. Budapest University of Technology and Economics Department of Applied Biotechnology and Food Science, Műegyetem rkp. 3. H-1111 Budapest, Hungary
2. CycloLab Cyclodextrin R&D Laboratory Ltd., Illatos u. 7. H-1097 Budapest, Hungary

Corresponding author: molnar.monika@vbk.bme.hu

Table S1 The population growth [optical density, OD] in the presence of the tested α- and β-CD concentrations, in the *P. aeruginosa* DSM 1117 large volume model system. Significant stimulation are marked by bold italics.

	Optical density [-] 72 h	
Concentration [mM]	ACD	BCD
Control	2.454 ± 0.039	2.109 ± 0.034
1	2.417 ± 0.056	2.182 ± 0.061
5	***2.873 ± 0.035***	***2.350 ± 0.053***
10	***3.149 ± 0.097***	2.093 ± 0.077

Table S2 The population growth [optical density, OD] in the presence of the tested α- and β-CD derivatives concentrations, in the *P. aeruginosa* PAO1 small volume model system. Significant effects compared to control (inhibition or stimulation) are marked by bold italics.

**α-CD and its derivatives**		Optical density [-] 24 h	48 h
Control		0.74 ± 0.03	0.98 ± 0.05
ACD [mM]	0,5	0.74 ± 0.01	***0.94 ± 0.02***
	2,5	0.74 ± 0.02	***0.93 ± 0.01***
	12,5	***0.67 ± 0.02***	***0.89 ± 0.02***
Control		0.74 ± 0.03	0.98 ± 0.05
QAACD [mM]	0,5	0.78 ± 0.01	0.95 ± 0.01
	2,5	0.76 ± 0.03	***1.05 ± 0.03***
	12,5	***0.68 ± 0.01***	0.98 ± 0.02
Control		0.74 ± 0.02	0.96 ± 0.05
HPACD [mM]	0,5	0.73 ± 0.01	0.91 ± 0.02
	2,5	0.68 ± 0.01	***0.87 ± 0.02***
	12,5	***0.60 ± 0.03***	0.96 ± 0.07
Control		0.98 ± 0.02	0.96 ± 0.05
ACDPS [mM]	0,5	0.97 ± 0.02	0.97 ± 0.05
	2,5	***0.88 ± 0.02***	***1.16 ± 0.01***
	12,5	***0.89 ± 0.02***	***1.24 ± 0.05***

**β-CD and its derivatives**		Optical density [-] 24 h	48 h
Control		0.71 ± 0.02	0.88 ± 0.02
BCD [mM]	0,5	***1.04 ± 0.01***	***1.23 ± 0.02***
	2,5	***0.90 ± 0.02***	***1.01 ± 0.02***
	12,5	***1.00 ± 0.03***	***1.17 ± 0.02***
Control		0.98 ± 0.02	0.96 ± 0.05
QABCD [mM]	0,5	0.98 ± 0.01	0.94 ± 0.02
	2,5	0.97 ± 0.02	0.92 ± 0.02
	12,5	***0.87 ± 0.01***	***1.22 ± 0.02***
Control		0.74 ± 0.02	0.96 ± 0.05
HPBCD [mM]	0,5	0.76 ± 0.02	0.95 ± 0.06
	2,5	***0.64 ± 0.01***	0.78 ± 0.01
	12,5	***0.54 ± 0.04***	***0.72 ± 0.03***
Control		0.98 ± 0.02	0.96 ± 0.05
BCDPS [mM]	0,5	0.93 ± 0.07	***0.85 ± 0.07***
	2,5	0.89 ± 0.03	0.89 ± 0.03
	12,5	***0.77 ± 0.01***	***1.15 ± 0.03***
Control		0.74 ± 0.03	0.98 ± 0.05
RAMEB [mM]	0,5	0.76 ± 0.02	0.94 ± 0.02
	2,5	0.74 ± 0.01	0.96 ± 0.02
	12,5	***0.68 ± 0.02***	***0.87 ± 0.03***
Control		0.74 ± 0.02	0.96 ± 0.05
SBEBCD [mM]	0,5	0.74 ± 0.01	0.87 ± 0.02
	2,5	0.67 ± 0.01	0.90 ± 0.04
	12,5	***0.54 ± 0.05***	0.83 ± 0.04

Table S2 The population growth [optical density, OD] in the presence of the tested α- and β-CD concentrations, in the *P. aeruginosa* DSM 1117 large volume model system. Significant stimulation are marked by bold italics.

	Optical density [-] 72 h	
Concentration [mM]	ACD	BCD
Control	2.454 ± 0.039	2.109 ± 0.034
1	2.417 ± 0.056	2.182 ± 0.061
5	***2.873 ± 0.035***	***2.350 ± 0.053***
10	***3.149 ± 0.097***	2.093 ± 0.077
